# Early neurological signs in infants identified through neonatal screening for SMA: do they predict outcome?

**DOI:** 10.1007/s00431-024-05546-y

**Published:** 2024-04-18

**Authors:** Marika Pane, Giulia Stanca, Chiara Ticci, Costanza Cutrona, Roberto De Sanctis, Matteo Pirinu, Giorgia Coratti, Concetta Palermo, Beatrice Berti, Daniela Leone, Michele Sacchini, Margherita Cerboneschi, Lavinia Fanelli, Giulia Norcia, Nicola Forcina, Anna Capasso, Gianpaolo Cicala, Laura Antonaci, Martina Ricci, Maria Carmela Pera, Chiara Bravetti, Maria Alice Donati, Elena Procopio, Emanuela Abiusi, Alessandro Vaisfeld, Roberta Onesimo, Francesco Danilo Tiziano, Eugenio Mercuri

**Affiliations:** 1 Centro Pediatrico Nemo and Pediatric Neurology Unit, Fondazione Policlinico “A. Gemelli” IRCCS, Largo Agostino Gemelli, 8, 00168 Rome, Italy; 2 Pediatric Neurology Unit, Università Cattolica del Sacro Cuore, Largo Francesco Vito, 1, 00168 Rome, Italy; 3 Metabolic and Muscular Unit, Meyer Children’s Hospital, Viale Gaetano Pieraccini, 24, 50139 Florence, Italy; 4 Rehabilitation Unit, Meyer Children’s Hospital, Viale Gaetano Pieraccini, 24, 50139 Florence, Italy; 5 Department of Life Sciences and Public Health, Section of Genomic Medicine, Fondazione Policlinico “A. Gemelli” IRCCS – Università Cattolica del Sacro Cuore, Largo Agostino Gemelli, 8, 00168 Rome, Italy; 6 Department of Pediatrics, Fondazione Policlinico Universitario “A. Gemelli” IRCCS, Largo Agostino Gemelli, 8, 00168 Rome, Italy

**Keywords:** Newborn screening, SMA, Floppy infant module, Neonatal neurological examination

## Abstract

Neonatal screening for SMA has allowed the identification of infants who may present with early clinical signs. Our aim was to establish whether the presence and the severity of early clinical signs have an effect on the development of motor milestones. Infants identified through newborn screening were prospectively assessed using a structured neonatal neurological examination and an additional module developed for the assessment of floppy infants. As part of the follow-up, all infants were assessed using the HINE-2 to establish developmental milestones. Only infants with at least 24 months of follow-up were included. Normal early neurological examination (*n* = 11) was associated with independent walking before the age of 18 months while infants with early clinical signs of SMA (*n* = 4) did not achieve ambulation (duration follow-up 33.2 months). Paucisymptomatic patients (*n* = 3) achieved ambulation, one before the age of 18 months and the other 2 between 22 and 24 months.

*  Conclusion*: Our findings suggest that early clinical signs may contribute to predict motor milestones development.

**Table:** 

**What is Known:** *• There is increasing evidence of heterogeneity among the SMA newborns identified via NBS.* *• The proposed nosology describes a clinically silent disease, an intermediate category (‘paucisymptomatic’) and ‘symptomatic SMA’.*	
**What is New:** *• The presence of minimal clinical signs at birth does not prevent the possibility to achieve independent walking but this may occur with some delay.* *• The combination of genotype at SMN locus and clinical evaluation may better predict the possibility to achieve milestones.*

**What is Known:**

*• There is increasing evidence of heterogeneity among the SMA newborns identified via NBS.*

*• The proposed nosology describes a clinically silent disease, an intermediate category (‘paucisymptomatic’) and ‘symptomatic SMA’.*

**What is New:**

*• The presence of minimal clinical signs at birth does not prevent the possibility to achieve independent walking but this may occur with some delay.*

*• The combination of genotype at SMN locus and clinical evaluation may better predict the possibility to achieve milestones.*

## Introduction

Following the availability of disease modifying treatment, neonatal screening (NBS) for spinal muscular atrophy (SMA) has recently become available in several countries, with increasing evidence of clinical and genetic heterogeneity among the newborns identified via NBS [1]. Finkel and Benatar recently proposed a conceptual framework for a possible classification of these infants [2]. The proposed nosology describes a clinically silent disease, with clinically normal infants and, at the other end of the spectrum, ‘symptomatic SMA’ with infants already presenting typical clinical findings. An intermediate category, ‘prodromal disease’ includes infants who have subtle symptoms. These patients have also been described as ‘paucisymptomatic’ or ‘oligosymptomatic’.

The new classification mirrors the results of our recent clinical study based on structured neurological examinations of infants identified through NBS [3]. Our study confirmed that in addition to the infants who already show obvious clinical signs of SMA at birth or soon after birth (type 0 and type 1.1 or 1A), others may have relatively minor clinical signs, such as mild hypotonia, weak/absent reflexes and tongue fasciculations.

The proposed nosology allows to overcome some of the ambiguity related to the term ‘presymptomatic’ that until recently was used to describe all the infants, identified via NBS or because of a positive family history, who did not have the full clinical signs of SMA. This is also obvious when reviewing the early clinical trials in ‘presymptomatic’ patients in which a number of cases were included even if they had subtle neurological signs [4].

The identification of infants with minimal signs, consistent with the prodromic phase described by Finkel and Benatar [2], has raised the question of whether the possible prognostic value of those signs. All the clinical studies on ‘presymptomatic’ infants have shown that all infants treated before the onset of full clinical signs have a remarkable motor development but also report some variability. Both clinical trials and real world data report that a number of infants performing relatively less well than others suggesting that the differences may be related to the presence of early neurological signs [5, 6]. No systematic attempt using a structured neurological assessment has been made to establish whether the different outcome may be related to the possible presence of early clinical signs that may suggest that some infants were already in the prodromic phase.

As follow-up of our previous study [3], we had the opportunity to follow 18 patients for at least 24 months. The aim of this paper was to establish the outcome in this cohort in relation to their initial clinical neurological signs.

## Material and methods

The infants were identified in a pilot study on the feasibility of NBS in two large Italian regions (Lazio and Toscana) and prospectively enrolled between Sept 2019 and October 2021. The confirmation of the diagnosis and the molecular prognosis was always obtained before the age of 2 weeks. As part of our protocol, infants were assessed using a structured neonatal neurological examination (HNNE) and an additional module developed for the assessment of floppy infants [7]. As part of the follow-up, all infants were assessed using the HINE-2, a proforma specifically designed to assess developmental milestones [8] that were always accurately reported also with the support of videos from the families. Only infants with at least 24 months of follow-up were included. The Shapiro–Wilk test of normality was used to establish normal distribution of continuous variables.

All the assessments were performed by MP and BB in Rome and by MS in Florence.

## Results

The cohort includes all the 18 infant identified by newborn screening in the time frame of the study. Nine had 2 *SMN2* copies (one also having the heterozygous c.859G > C *SMN2* modifier variant), 3 had 3 copies, 4 had 4 copies and 2 more than 4 copies.

### Neonatal assessment

All newborns were assessed at the time of the first consultation (date of examination range 3–13 days after birth). Clinical neurological signs at birth suggestive of SMA were found in 4 patients, all with 2 *SMN2* copies.

Minimal signs, consistent with the label of paucisymptomatic and prodromic phase, were found in three infants. Two had 2 copies of *SMN2* and one 3 copies.

The remaining 11 all had a normal examination. *SMN2* copies ranged from 2 to more than 4 copies.

Fourteen of the 18 patients were treated soon after diagnosis. In the remaining 4, all with SMN2 ≥ 4 copies, no treatment was initiated.

### Follow-up

**Fig. 1:**
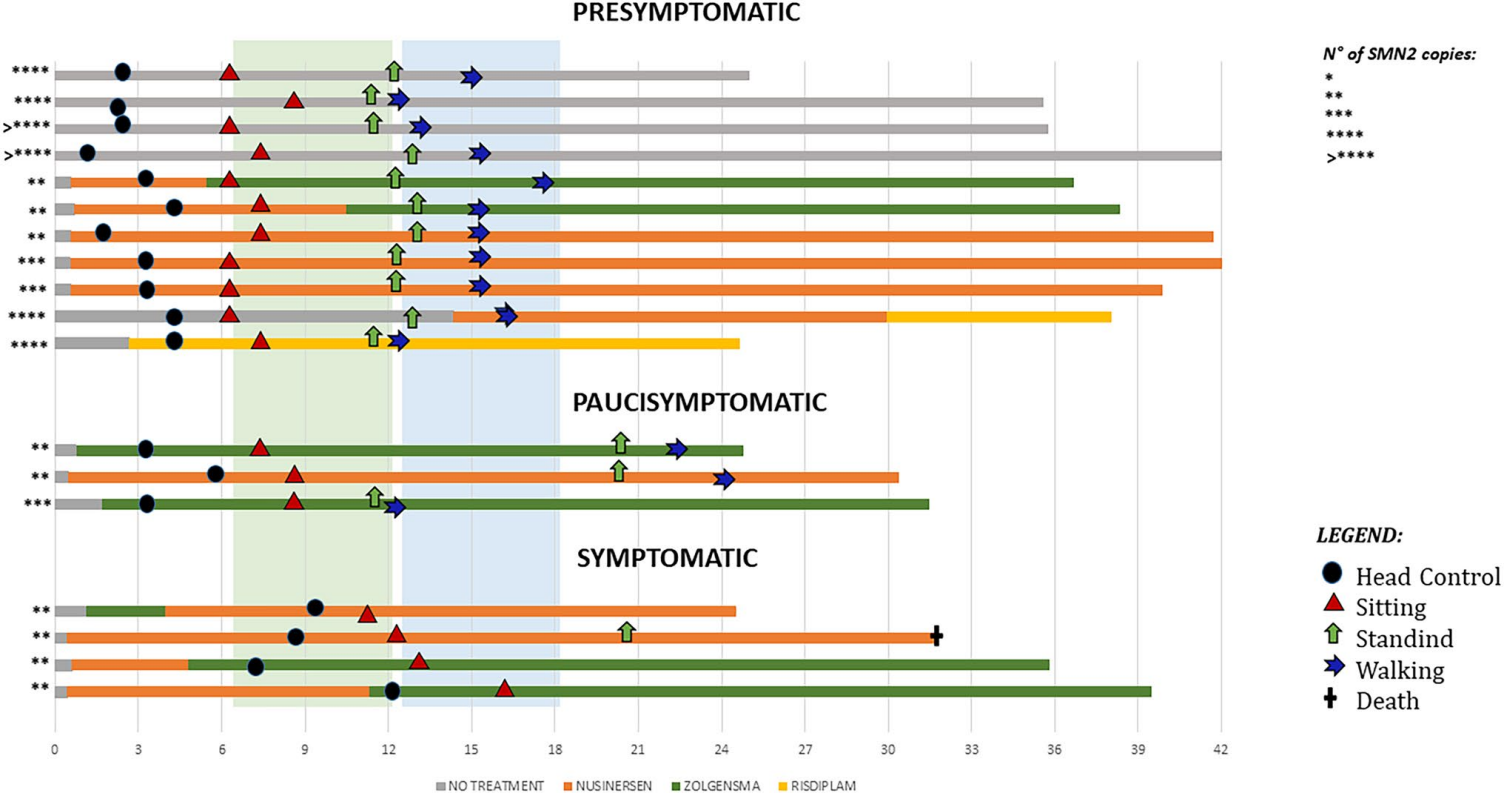
Details of the milestones achieved and of treatment

All the infants were followed at least for 24 months (mean duration follow-up 33.2, SD 6.7) with assessments at 6, 12, 18 and 24 months, and every 6 months after that. Figure 1 shows details of the milestones achieved and of treatment.

The four infants with obvious symptoms at birth, all treated, acquired the ability to sit unsupported (after the age of 9 months) and one acquired the ability to stand. The child who developed the ability to stand died at the age of 33 months for aspiration pneumonia partly also in relation to the refusal of the family to comply with standards of care. The remaining 3, last seen at the age of 24, 36 and 40 months, had not acquired the ability to walk.

All three paucisymptomatic patients, all treated, acquired the ability to sit between 6 and 9 months. All three also acquired the ability to walk unsupported, one (with 3 *SMN2* copies) at 12 months, and the other 2 (both with 2 *SMN2* copies) after 18 months.

All the 11 asymptomatic patients acquired the ability to sit independently around the age of 6 months and to walk independently between 12 and 18 months. This included the 4 infants with 4 or more copies who had not been treated while all the other patients received treatment (see Fig. 1).

## Discussion

We report follow-up data in a small cohort of infants with SMA identified through NBS who were systematically examined in the neonatal period and were followed for at least 24 months. The use of a structured neonatal neurological examination at the time of diagnosis allowed us to classify the small cohort into subgroups according to the presence and severity of clinical signs and to explore if these were related to the neuromotor development. The analysis of the results, even if limited by the cohort size, suggests that the patterns of development in these subgroups may be different.

Those who already had obvious signs of SMA all achieved sitting but none achieved independent walking, as previously reported in symptomatic patients [9, 10], despite the early genetic diagnosis allowed treatment at an earlier age compared to the symptomatic patients in clinical trials [11, 12] or in real world setting. This may be partly explained by the fact that, being already symptomatic at birth, they had the more severe phenotype with neonatal onset (1.1 or 1A) that was excluded in most clinical trials [11, 12].

At the other end of the spectrum, all infants who had a completely normal neurological examination at the time of diagnosis achieved sitting and independent walking within the time frame used by the WHO to define these milestones in typically developing children. Despite the limited number, our results in paucisymptomatic infants, in the prodromic phase, showed that all achieved independent walking, but in two of the three this was achieved after the age of 18 months with a mild delay compared to the asymptomatic infants. It is of note that the two children with a delay had 2 *SMN2* copies while the one who achieved walking within 18 months had 3 copies. Having 2 *SMN2* copies however was not the only determinant of outcome as 2 copies were also found in asymptomatic infants who had no delay and in some severely symptomatic patients who never achieved sitting. With the exceptions of 4 infants with 4 or more copies, all the others received treatment.

Although the limited size of our cohort does not allow to draw any definite conclusion, our findings suggest that the presence of minimal clinical signs at birth does not prevent the possibility to achieve independent walking but this may occur with some delay.

Our findings also suggest that the combination of genotype at SMN locus and clinical evaluation may better predict the possibility to achieve milestones and the time when are achieved than each of them individually. Further studies in larger cohorts will allow to perform a meaningful statistical analysis on a possible correlation between treatments and motor acquisitions that could not be performed in our cohort because of the small numbers in two of the three subgroups. Larger, more detailed studies are also needed to explore the possible additional contribution of biomarkers and neurophysiology.

## Data Availability

No datasets were generated or analysed during the current study.

## Abbreviations

HINE-2: Hammersmith Infant Neurological Examination—2
HNNE: Hammersmith Neonatal Neurological Examination
NBS: Newborn screening
SMA: Spinal muscular atrophy
SMN: Survival motor neuron
